# Enrichment of Human Dermal Stem Cells from Primary Cell Cultures through the Elimination of Fibroblasts

**DOI:** 10.3390/cells12060949

**Published:** 2023-03-21

**Authors:** Christin Starzonek, Mouna Mhamdi-Ghodbani, Stefan Henning, Marc Bender, Sarah Degenhardt, I-Peng Chen, Mohamed Said, Rüdiger Greinert, Beate Volkmer

**Affiliations:** 1 Skin Cancer Center, Division of Molecular Cell Biology, Elbe Kliniken Stade-Buxtehude, 21614 Buxtehude, Germany; 2 Pediatric Practice, 22587 Hamburg, Germany

**Keywords:** dermal stem cell, DSC, fibroblast, Geneticin, selective detachment, immunomagnetic cell separation, MACS^®^, EasySep™, positive selection, negative selection

## Abstract

Dermal stem cells (DSCs), which are progenitor cells of melanocytes, are isolated from human foreskin and cultivated as mixed cultures containing both DSCs and fibroblasts in varying proportions. These contaminating fibroblasts may have an impact on the results of experimental studies and are a serious limitation for certain applications. The aim of the present study was to purify or enrich DSCs—an indispensable step towards future investigations. Applying different methods, we demonstrated that highly enriched DSCs with a good recovery rate can be obtained through positive selection with MACS^®^ immunomagnetic cell sorting. These DSCs remain vital and proliferate constantly in culture, maintaining a high level of purity after enrichment. Other approaches such as treatment with Geneticin or selective detachment were not suitable to purify DSC-fibroblast co-cultures. Overall, enriched DSCs represent a novel and unique model to study the effects of UV radiation on the differentiation of DSCs into melanocytes and their potential relevance in the genesis of malignant melanoma.

## 1. Introduction

Melanoma is the most aggressive and most lethal form of skin cancer. The identity of the original target cell, which acquires the requisite DNA lesions for transformation into melanoma, still remains elusive. In human skin, neural crest-derived precursors of melanocytes, dermal stem cells (DSCs), are seen to be involved in the origin of melanoma [1,2]. DSCs can be isolated from human foreskin and cultivated as primary cell culture in specific stem cell mediums [3]. However, these DSC cultures contain a considerable number of fibroblasts [4] due to the cultivation of all isolated dermal cells and the low percentage of DSCs in the skin. It is well known that contaminating fibroblasts with a high proliferative potential can lead to the overgrowth of the desired cell population, even if the initial fibroblast contamination is low [5,6]. More importantly, their presence causes difficulties in certain analyses where both cell types are indistinguishable, e.g., the analysis of epigenetics, gene expression, protein expression, or exosomes. For the proper study of DSCs, it is indispensable to work with pure or enriched stem cell cultures. Therefore, establishing a method to eliminate this undesired cell population prior to performing experiments is paramount. Several attempts to eliminate unwanted fibroblasts from cell cultures have been previously described for different cell lines/types. Traditional methods include (i) the addition of antimitotic drugs such as cytosine arabinoside (Ara-C) [7,8] or antibiotics such as Geneticin (G418) [9,10] into the cell culture medium, (ii) differential adhesion [11,12] and selective detachment methods [13,14], (iii) culturing in media without serum [13,15], (iv) immunomagnetic separation based on differential cell surface antigens [6,10,16], and (v) fluorescence-activated cell sorting (FACS) combining multiple parameters such as different surface markers, size, and granularity [17,18,19,20].

Here, we report an experimental study of three methods to enrich DSCs in fibroblast-containing primary cell cultures obtained from human foreskin. We decided to examine Geneticin treatment and selective detachment due to simplicity and availability reasons, and additionally chose immunomagnetic cell sorting for higher specificity. The methods are as follows.

(a) The elimination of fibroblasts through addition of Geneticin to the culture medium. The aminoglycoside antibiotic Geneticin is commonly used to select transfected cells. It inhibits protein synthesis by blocking the elongation step in both prokaryotic and eukaryotic cells, thereby interfering with cell growth and proliferation. Due to known differential cytotoxic effects on eukaryotic cells with different growth rates, it is also used for the elimination of contaminating fibroblasts from mixed cultures. Cytotoxicity is observed after 1–2 cell divisions, with the highest impact on the most rapidly dividing cells. Further action of Geneticin on cells can include caspase-3-dependent apoptosis, leading to cell death. This selective elimination method has been successfully described for melanocyte cultures from various tissues containing fibroblasts [21].

(b) The segregation of DSCs and fibroblasts via selective detachment with Accutase™ or trypsin-EDTA. Selective detachment can be used when different cell populations in adherent growing cell cultures possess varying detachment properties, so that they are selectively released at different times of incubation. Depending on the specific cell culture, with this method, either fibroblasts are detached first and can be removed with the supernatant [13], or the desired cells are released earlier while fibroblasts remain attached due to stronger adhesion [14]. Selective detachment with trypsin-EDTA is an established method for HDMEC (human dermal microvascular endothelial cells) contaminated with fibroblasts [14]. In studies with human corneal epithelial cells (CEC), the usage of cold trypsin-EDTA was also effective in separating fibroblasts. However, this method required multiple treatments, leading to senescence of CECs over time [13].

(c) The separation of the two cell types through immunomagnetic cell sorting based on CD90 or NGFRp75 (low-affinity nerve growth factor receptor, CD271). Magnetic cell sorting is a well-established method for the separation of various cell populations based on specific cell surface antigens and therefore allows for the enrichment of a particular cell population. In principle, specific antibodies bound to magnetic beads capture cells, which express the corresponding epitope. Using a magnetic field, the labeled cells are retained and therefore separated from unlabeled cells. Cells can be isolated via negative selection (labeling of undesired cells) or positive selection (labeling of target cells), depending on the particular antigen that is used [22,23]. We chose the fibroblast marker CD90 and the stem cell marker NGFRp75 (CD271) for negative and positive selections, respectively, because these are well-described, cell-specific markers [2,5,6] on which a large part of the commercially available selection kits is based on. Negative selection using magnetic beads conjugated to a CD90 antibody has been applied by Saalbach et al. to purify human dermal microvascular endothelial cells (HDMEC) that were experimentally contaminated with fibroblasts to a variable extent (5–50%) [5]. Similarly, human umbilical vein endothelial cell (HUVEC) cultures were enriched via negative selection (≥90%) and remained pure over multiple passages [13]. The positive selection of cell cultures can be utilized when suitable cell surface markers are expressed on the target cells. In primary cell culture, HDMECs can be separated from contaminating fibroblasts based on the labeling of CD31 on the surface of HDMECs [16]. Peng et al. showed the purification of nerve-derived human Schwann cell cultures containing fibroblasts by targeting NGFRp75 [6]. Likewise, human melanocyte precursors can be enriched from limbal tissue using positive (CD117/c-Kit) or negative (CD326/EpCAM and D7-FIB) selection, removing contaminating epithelial cells and fibroblasts [10]. In all three studies, the positively selected cells remained viable and functional and could subsequently be used for downstream applications. Two widely used immunomagnetic separation techniques are MACS^®^ (magnetic-activated cell sorting) technology from Miltenyi Biotec (Bergisch Gladbach, Germany) and EasySep™ technology from StemCell Technologies (Vancouver, BC, Canada).

The aim of this study was to obtain pure or enriched populations of DSCs—with a desired purity of at least 85% or more of the total cell population—for further studies exploring the genetic and epigenetic effects of UV irradiation on these cells.

## 2. Materials and Methods

### 2.1. Isolation and Cultivation of Cells

DSCs were isolated from human foreskins as previously described [3,4]. After dissociation of the dermis, whole dermal cells were seeded at a density of 6–8 × 10^5^ cells/mL in StemPro hESC SFM medium (Gibco, Waltham, MA, USA) in suspension culture flasks to be cultivated in spheres. After 12–14 days, the dermal spheres were dissociated with Accutase™ (Capricorn, Ebsdorfergrund, Germany) for 1 h at 37 °C. The proportion of DSCs in culture was measured via flow cytometry using the stem cell marker NGFRp75, and cells were seeded in Geltrex™-coated cell culture vessels (Geltrex™ LDEV-Free Reduced Growth Factor Basement Membrane Matrix, Gibco) for proliferation and subsequent experiments. Depending on the number of cells, experiments were conducted with single donor cell strains or a pool of multiple donor cell strains.

### 2.2. Doubling Time

Dissociated DSC-fibroblast co-cultures were seeded in Geltrex™-coated cell culture dishes (8.9 × 10^3^ cells/cm^2^) in StemPro hESC SFM medium. Starting the following day, cells were detached with Accutase™ at four time points with an interval of 24 h each. Cell counts of biological replicates, including technical triplicates, were measured using flow cytometry. Additionally, the proportion of DSCs in culture was determined via the flow cytometric staining of NGFRp75 to distinguish between DSCs and fibroblasts. The natural logarithm of the cell number was plotted against time, and a linear fit was applied for the exponential growth phase. Using the growth rate µ, which corresponds to the slope of the fit, the doubling time was calculated with the formula $t_{d}=\frac{\ln2}{µ}$.

### 2.3. Geneticin Treatment

Dissociated DSC-fibroblast co-cultures were seeded on Geltrex™-coated coverslips (13 mm Ø; VWR, Radnor, PA, USA) placed in 24-well plates (Greiner Bio-One, Kremsmünster, Austria) (5.0–5.7 × 10^4^ cells/coverslip) or in Geltrex™-coated cell culture dishes (3.1–3.9 × 10^4^ cells/cm^2^) in StemPro hESC SFM medium. Starting on the following day (day 0), cultures were treated with different concentrations of 50 or 100 µg/mL Geneticin (Gibco) for a total of two days. Then, cells were washed with phosphate-buffered saline (PBS^−/−^), fresh medium without Geneticin was added (day 2), and cells were monitored for a total of six days. Control cells were treated in the same way but without the addition of Geneticin. On days 0, 3, and 6 of the experiment, the total number of cells cultured in Petri dishes was measured, and cells grown on coverslips were fixed for subsequent immunofluorescence staining to determine the proportion of DSCs in culture.

### 2.4. Selective Detachment

Dissociated DSC-fibroblast co-cultures were seeded in Geltrex™-coated cell culture dishes (2.6–3.1 × 10^4^ cells/cm^2^) in StemPro hESC SFM medium. At 80–90% confluency, cells were washed with PBS^−/−^ and incubated with detachment reagents Accutase™ or trypsin-EDTA (0.25%/0.02%) (Merck, Darmstadt, Germany) for different periods. At the respective time points, the culture dish was tapped slightly and the supernatant with the detached cells was collected. The remaining and still attached cells were subsequently further detached, reaching a total incubation time of 5 min. For detachment with Accutase™, cells were collected at 1, 2, and 3 min time points. Time points for the trypsin-EDTA treatment were 0.5, 1, and 2 min. Control cells were incubated for 5 min. Cell counts of the different samples were measured, and the proportion of DSCs in culture was determined via the flow cytometric staining of NGFRp75 and CD90. Cell viability was assessed with propidium iodide staining.

### 2.5. Immunomagnetic Separation

DSC-fibroblast co-cultures were subjected to both positive or negative selection, while also comparing EasySep™ column-free and MACS^®^ automatic column-based separation methods. Magnetic bead separation was performed on fresh co-cultures grown on Geltrex™-coated cell culture vessels prior to selection to allow the cells to proliferate and to obtain sufficient amounts of cells. DSC-fibroblast co-cultures were dissociated with Accutase™ for 5 min, and single-cell suspensions were subjected to separation protocols. The cell counts were determined before and in both fractions after separation. Recovery was calculated as the ratio of the absolute number of DSCs in the purified fraction to the absolute number of DSCs in the initial sample.

#### 2.5.1. EasySep™ Column-Free Separation

The EasySep™ column-free separation of fibroblasts and DSCs was performed using 5 mL polystyrene round-bottom tubes (Corning, Corning, NY, USA), the EasyEights™ EasySep™ Magnet, and EasySep™ Selection Kits from StemCell Technologies. PBS^−/−^ containing 2% fetal bovine serum (FBS) (Capricorn) and 1 mM EDTA was used as selection buffer.

For EasySep™ negative selection (labeling of fibroblasts), the EasySep™ Human PE Positive Selection Kit II (cat. #17664) in combination with a PE-conjugated anti-CD90 antibody (clone 5E10, cat. #60045PE; both from StemCell Technologies) was used according to the manufacturer’s protocol, with slight modifications. Modifications of the kit protocol included: up to 1 × 10^7^ cells were resuspended in 1 mL of buffer (instead of 1 × 10^8^ cells/mL); 100 µL FcR blocker/mL of sample and 20 µL of PE-conjugated anti-CD90 antibody/mL of sample meaning 1 µg/mL antibody (recommended concentration 0.3–3 µg/mL) were added and incubated for 15 min at RT; wash step with centrifugation at 200× *g* for 10 min and resuspending of cell pellet in 1 mL buffer; for an optimal ratio of the selection cocktail to antibody, 50 µL of selection cocktail/mL of sample (instead of 100 µL/mL) were added and incubated for 15 min at RT; due to the high amount of fibroblasts to be captured, 100 µL of RapidSpheres™ (beads)/mL of sample (instead of 75 µL/mL) were added and incubated for 10 min at RT; sample was topped up with buffer to 2.5 mL and placed into the magnet for 10 min; supernatant was carefully pipetted off and transferred to a new tube; cells left in the tube were resuspended in 2.5 mL buffer and the isolation step in the magnet was repeated with both the supernatant and the resuspended beads to a total of 2–3 separations.

For EasySep™ positive selection (labeling of DSCs), the EasySep™ Human CD271 Positive Selection Kit II (cat. #17849; StemCell Technologies) was used according to the manufacturer’s protocol, with slight modifications: approximately 1–2 × 10^7^ cells were resuspended in 1 mL of buffer (instead of 1 × 10^8^ cells/mL); 25 µL of FcR blocker/mL of sample as well as 50 µL of selection cocktail/mL of sample were added and incubated for 15 min at RT; 50 µL of RapidSpheres™ (beads)/mL of sample were added and incubated for 5 min at RT; sample was topped up with buffer to 2.5 mL and placed into the magnet for 5 min; supernatant was carefully pipetted off and transferred to a new tube; cells left in the tube were resuspended in 2.5 mL buffer and the isolation step in the magnet was repeated to a total of 3–4 separations.

The supernatant was referred to as the negative fraction. The leftover CD90-PE- or CD271(NGFRp75)-labeled cells bound to the magnetic beads were referred to as the positive fraction. These two cell suspensions obtained from magnetic separation were collected for immunostaining via flow cytometry and further propagation.

#### 2.5.2. MACS^®^ Automatic Column-Based Separation

The MACS^®^ automatic column-based separation was conducted with 5 mL polystyrene round-bottom tubes (Corning), the autoMACS^®^ Pro Separator, and MACS^®^ MicroBeads from Miltenyi Biotec. PBS^−/−^ containing 0.5% BSA and 2 mM EDTA was used as selection buffer. The buffer was prepared by diluting MACS^®^ BSA Stock Solution (cat. #130-091-376) 1:20 with autoMACS^®^ Rinsing Solution (cat. #130-091-222; both from Miltenyi Biotec). Before starting the separation protocols, dissociated cell suspensions were filtered through 30 µm filters (cat. #130-041-407; Miltenyi Biotec) to remove cell clumps that could clog the columns, and DNase digestion was performed on the cell suspension. Cells were treated with 200 U/mL DNase I (cat. #18535.01; Serva, Heidelberg, Germany) diluted in 5 mL of Dulbecco’s modified Eagle medium (DMEM) (Gibco) containing 10% FBS (Capricorn) for 5 min at RT. The samples were washed with 15 mL of DMEM and subjected to separation. Cells and buffer were kept cold (4 °C) throughout the separation protocol.

Only once (see Supplementary Materials Figure S1) was the removal of non-specific binding material conducted before separation using Basic MicroBeads (cat. #130-048-001, Miltenyi Biotec) according to the manufacturer’s protocol: up to 1 × 10^7^ cells were resuspended in 50 µL of buffer and 5 µL of pre-diluted (1:10) Basic MicroBeads/1 × 10^7^ cells were added; incubation for 15 min at 4 °C; wash step by adding 2 mL of buffer/1 × 10^7^ cells and centrifugation at 300× *g* for 5 min; cell pellet (up to 1 × 10^8^ cells) was resuspended in buffer to a total volume of 500 µL, and sample was placed into the tube rack in the autoMACS^®^ Pro Separator; the sensitive single-column depletion program *DepleteS* and the wash program *Rinse* were selected. The negative fraction containing unbound cells was subjected to separation protocols. All other separations shown in the results section were conducted without the prior use of Basic MicroBeads.

For MACS^®^ negative selection (labeling of fibroblasts), two different MACS^®^ MicroBeads were tested.

(a) Anti-PE MicroBeads (cat. #130-048-801) in combination with a PE-conjugated anti-CD90 antibody (clone DG3, cat. #130-117-388; both from Miltenyi Biotec) were used according to the manufacturer’s protocol, with slight modifications. Modifications of the MicroBeads protocol included: up to 1 × 10^7^ cells were resuspended in buffer to a total volume of 200 µL (instead of 100 µL/1 × 10^7^ cells); 16 µL of PE-conjugated anti-CD90 antibody/1 × 10^7^ cells meaning 8 µg/mL antibody (instead of 2 µL/1 × 10^7^ cells meaning 2 µg/mL) were added and incubated for 10 min at 4 °C in the dark; wash step with centrifugation at 300× *g* for 5 min and resuspending of cell pellet in buffer to a total volume of 120 µL (instead of 80 µL/1 × 10^7^ cells); a four-fold increase in the amount of magnetic beads was incorporated to attempt to capture more anti-CD90 bound fibroblasts: 80 µL of Anti-PE MicroBeads/1 × 10^7^ cells (instead of 20 µL/1 × 10^7^ cells) were added and incubated for 15 min at 4 °C in the dark; wash step with centrifugation at 300× *g* for 5 min; cell pellet was resuspended in 1 mL of buffer (instead of 500 µL), and sample was placed into the tube rack in the autoMACS^®^ Pro Separator; the single-column depletion program *Deplete* and the wash program *Rinse* were selected.

(b) CD90 MicroBeads (cat. #130-096-253; Miltenyi Biotec) were used according to the manufacturer’s protocol, with slight modifications: up to 1 × 10^7^ cells were resuspended in buffer to a total volume of 20 µL (instead of 80 µL/1 × 10^7^ cells); a four-fold increase in the amount of magnetic beads was incorporated to attempt to capture more CD90 expressing fibroblasts: 80 µL of CD90 MicroBeads/1 × 10^7^ cells (instead of 20 µL/1 × 10^7^ cells) were added and incubated for 15 min at 4 °C in the dark; wash step with centrifugation at 300× *g* for 5 min; cell pellet was resuspended in 1 mL of buffer (instead of 500 µL), and sample was placed into the tube rack in the autoMACS^®^ Pro Separator; the single-column depletion program *Deplete* (loading rate of 4 mL/min) or the more sensitive program *DepleteS* (loading rate of 1 mL/min), and the wash program *Rinse* were selected.

For MACS^®^ positive selection (labeling of DSCs), Neural Crest Stem Cell (NCSC) MicroBeads that are conjugated to anti-NGFRp75 antibodies (cat. #130-097-127; Miltenyi Biotec) were used according to the manufacturer’s protocol, with slight modifications: up to 1 × 10^7^ cells were resuspended in buffer to a total volume of 160 µL (instead of 80 µL/1 × 10^7^ cells); 40 µL of NCSC MicroBeads/1 × 10^7^ cells (instead of 20 µL/1 × 10^7^ cells) were added and incubated for 15 min at 4 °C in the dark; wash step with centrifugation at 300× *g* for 5 min; cell pellet was resuspended in 1 mL of buffer (instead of 500 µL), and sample was placed into the tube rack in the autoMACS^®^ Pro Separator; the double-column positive separation program *Posseld2* and the wash program *Rinse* were selected.

At the end of the separation process, the autoMACS^®^ Pro Separator collected two separate fractions. The negative cell fraction contained unlabeled cells, whereas the positive cell fraction included magnetically labeled cells. Finally, the fraction containing the enriched DSCs was further cultivated for around 11–12 days. The distribution of DSCs and fibroblasts in culture was measured via the flow cytometric staining of NGFRp75 and CD90 before, directly after separation, and after multiple days of cultivation to monitor whether fibroblasts were growing back in the purified DSC culture.

### 2.6. Immunostaining

Cells grown on glass coverslips were washed with PBS^−/−^ and then fixed with ice-cold methanol and acetone (both from Roth, Karlsruhe, Germany) for 5 min each at −20 °C. Slides were blocked with 3% bovine serum albumin (Roth) in PBS containing 0.9 mM Ca^2+^ and 0.5 mM Mg^2+^ (PBS^+/+^) followed by 5% goat serum (Dako, Glostrup, Denmark) in PBS^−/−^ both for 30 min at RT. Cells were incubated with 2 µg/mL rabbit anti-NGFRp75 antibody (clone EP1039Y, cat. #ab52987; Abcam, Cambridge, UK) and 1 µg/mL mouse anti-CD90 antibody (clone AS02, cat. #DIA100; Dianova, Hamburg, Germany) diluted in 3% bovine serum albumin (BSA) in PBS^+/+^ for 2 h at 37 °C. After washing the slides with PBS^−/−^, secondary goat antibodies conjugated to Alexa Fluor 488 or 594 (cat. #A11029 and A11012; Invitrogen, Waltham, MA, USA) were incubated for 1 h at 37 °C. Cells were counterstained with 0.1 µg/mL DAPI (Sigma, St. Louis, MO, USA) for 5 min and examined using a Leica DMR fluorescence microscope.

For flow cytometry analysis, cell samples were incubated with 0.3 µg/mL APC-conjugated recombinant anti-CD271 (LNGFR) antibody (clone REA844, cat. #130-112-602; Miltenyi Biotec) and 0.5 µg/mL PE-conjugated mouse anti-CD90 antibody (clone 5E10, cat. #60045PE; StemCell Technologies) diluted in 0.5% BSA/2 mM EDTA/PBS^−/−^ for 10 min at 4 °C. Next, 1 μg/mL propidium iodide (cat. #130-093-233; Miltenyi Biotec) was added to exclude dead cells prior to the measurement of at least 10,000 cells per sample using a Guava easyCyte 8HT flow cytometer (Merck). The proportion of CD271(NGFRp75)-positive cells represented the frequency of DSCs.

## 3. Results and Discussion

### 3.1. Stem Cell Frequency of DSC-Fibroblast Co-Cultures

**Figure 1:**
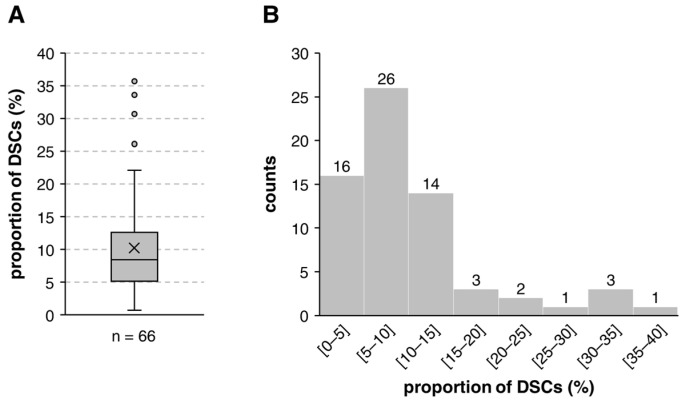
Frequency of stem cells in DSC-fibroblast co-cultures. Dermal cells were cultured in stem cell medium for up to two weeks to form spheres. The spheres were enzymatically dissociated with Accutase™, and the percentage of DSCs (NGFRp75-positive cells) in the culture was determined via flow cytometry. (**A**) Boxplot displaying the median with 25th/75th percentiles. The cross (×) inside the box indicates the mean DSC frequency. Dots represent outliers. (**B**) The histogram shows the abundance distribution of DSC frequencies of individual donor cell strains divided into ranges of 5%. Results from 66 donors.

After the first two weeks of cultivation in the stem cell medium, the proportion of DSCs in dissociated dermal spheres averaged only 10.2%, with a great donor-dependent variation ranging between 0.7% and 35.7% (Figure 1A). The majority of the donor cell strains (six out of seven, corresponding to 85%) displayed a stem cell frequency below 15% (Figure 1B). Higher proportions of DSCs were rare and did not occur frequently. During cultivation, the stem cell content may decrease or increase but was usually relatively stable and varied only within a small percentage range.

Thus, cultures of DSCs are particular cell cultures, which are more likely to be termed a co-culture of DSCs and fibroblasts (and maybe other dermal cells in a very small proportion) rather than a fibroblast-contaminated DSC culture. To eliminate the vast number of fibroblasts in these co-cultures, thereby enriching the amount of DSCs, three methods have been tested that are generally well described for several cell types but have not yet been applied to dermal stem cells.

### 3.2. Elimination of Fibroblasts with Geneticin

**Figure 2:**
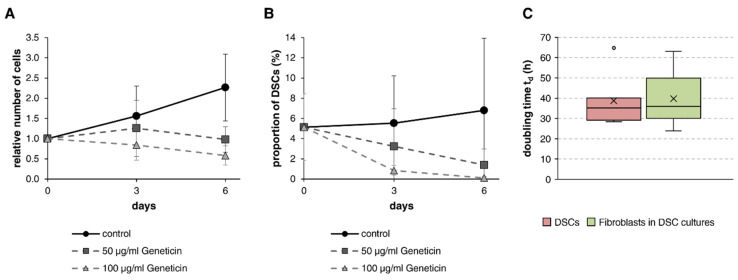
Geneticin treatment of DSC-fibroblast co-cultures. Dermal cells were cultured in StemPro hESC medium without Geneticin or with 50 µg/mL or 100 µg/mL Geneticin for two days, and cell growth was monitored for a total of six days. Number of total cells in relation to the cell count of control cells at day 0 (**A**) and frequency of DSCs in culture (**B**) were measured at days 0, 3, and 6. Values are presented as mean ± SDs. Sample size: n = 3. (**C**) Doubling time of DSCs and the respective fibroblasts in co-cultures without Geneticin treatment. Boxplot displaying the median with 25th/75th percentiles. The cross (×) inside the box indicates the mean DSC frequency. Dots represent outliers. Sample size: n = 7.

We performed treatment with Geneticin for the selective killing of fibroblasts in DSC-fibroblast co-cultures while also determining the growth rates of both DSCs and the respective fibroblasts in (non-Geneticin-treated) co-cultures. Geneticin treatment led to reduced cell densities visible after three days compared to control cells. While the untreated cells were nearly confluent on day six of the experiment, both Geneticin concentrations caused distinct growth inhibition or cell death as well as changes in cell morphology (Figure S2A–C). Control cells proliferated over the course of six days, leading to an average of 2.3 ± 0.8 times the number of cells compared to the beginning of the experiment, whereas cell cultures subjected to 50 µg/mL Geneticin showed stagnating cell counts of 1.0 ± 0.3. Samples treated with 100 µg/mL Geneticin even displayed steadily decreasing cell counts, resulting in a relative cell number of 0.6 ± 0.2 at the end of the experiment (Figure 2A). Immunofluorescent staining of the cell cultures revealed that Geneticin not only harmed fibroblasts but also dramatically affected the frequency of DSCs (Figure S2D). The proportion of DSCs decreased strongly from 5.1 ± 3.3% at day 0 to 1.4 ± 1.6% and 0.1 ± 0.1% with 50 µg/mL and 100 µg/mL Geneticin, respectively, until day six (Figure 2B). Instead of an enrichment of the stem cell proportion, treatment with Geneticin induced the opposite effect in the DSC-fibroblast co-cultures. The measurement of growth rates revealed that, on average, both cell types proliferated equally fast, with DSCs reduplicating every 38.8 h and fibroblasts showing a doubling time of 39.7 h (Figure 2C).

Various aspects need to be considered when comparing the effectiveness of Geneticin treatment in different cell cultures. The most important aspect is the proliferation rate of the various cell populations. In fact, DSCs and fibroblasts have similar growth rates in co-cultures in stem cell medium. This explains why both cell populations were similarly affected by Geneticin treatment. While the minor population of DCSs in culture died, some of the outnumbering fibroblasts survived the treatment. In contrast, Geneticin is known to be an effective eliminator of fibroblasts in melanocyte cultures. Melanocytes are slow-dividing cells that rarely proliferate in vivo. During in vitro cultivation, their doubling time varies from 48 h up to 10 days depending on the growth conditions [21]. Fibroblasts divide much faster with a doubling time ranging from 40–54 h [24], permitting an effective purification of melanocyte cultures since Geneticin acts on the most rapidly dividing cells. How cells respond to Geneticin also depends on their cell metabolism, with some cell lines/cell types being able to better tolerate this kind of cell stress. Melanocytes appear to be naturally resistant to Geneticin. Stem cells, by contrast—and as seen in this study, especially DSCs—seem to be particularly sensitive, explaining why Geneticin is not commonly applied to enrich stem cell cultures. Moreover, different compositions of the culture media (e.g., serum, glutamine, insulin) could influence the impact of Geneticin, impeding the transfer of an established procedure for one culture condition to another. Even though Geneticin is an effective and cheap method with minimal effort to eliminate fibroblasts from melanocyte cultures [10,21], it is not suitable to purify DSC cultures. Furthermore, one has to keep in mind that non-cell-specific agents such as Geneticin may also affect the proliferation, phenotype, and functional properties of the surviving cell population, possibly influencing downstream applications [19,20].

### 3.3. Segregation of DSCs and Fibroblasts via Selective Detachment

**Figure 3:**
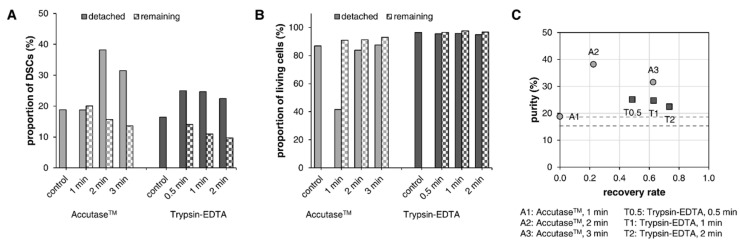
Selective detachment of DSC-fibroblast co-cultures with 10–20% stem cells. Dermal cells were incubated with Accutase™ for 1, 2, or 3 min or with trypsin-EDTA for 0.5, 1, or 2 min, and detached cells were collected. (**A**) Frequency of DSCs in samples measured using NGFRp75 staining. (**B**) Viability of total cells examined with propidium iodide. (**C**) Plot of recovery rate (*x*-axis) versus purity (*y*-axis) of detached cells at the individual incubation times. Purity: frequency of DSCs. Recovery rate: ratio of the absolute number of DSCs in the detached sample to the absolute number of DSCs before detachment. Dotted lines indicate the DSC frequency of control cells detached with Accutase™ (light gray) or trypsin-EDTA (dark gray). Results from one representative donor cell strain.

Analysis of DSC-fibroblast co-cultures with different stem cell proportions (<5%, 10–20%, >30%) revealed that fibroblasts seemed to adhere more strongly to the Geltrex™-coated cell culture vessel than DSCs. DSCs detached faster—with both Accutase™ and trypsin-EDTA—and were found at a higher proportion in the detached sample, while showing a decreasing frequency in the remaining sample (Figure 3A and Figure S3C). The use of Accutase™ led to the slower detachment of cells (Figure S3A) compared to detachment with trypsin-EDTA (Figure S3B). For Accutase™, an increase in DSC frequency in the detached sample was observed (ranging between 3.6–23.6 percentage points depending on the donor) at 2 min and 3 min of incubation. With trypsin-EDTA, the time points of 0.5 min and 1 min resulted in the strongest increase in DSC frequency in the detached sample, varying from 1.0–21.1 percentage points. Overall, detachment with Accutase™ led to higher DSC enrichment than trypsin-EDTA; however, interestingly, the control cells (total detachment after 5 min) also showed lower DSC frequencies when detached with trypsin-EDTA. Results from cell viability assessment (Figure 3B and Figure S3D) showed that more than 90% of the cells were viable in all samples (detached, remaining, and controls) treated with trypsin-EDTA. In contrast, Accutase™ slightly impaired cell viability in general, as seen in the controls, and incubation with Accutase™ for 1 min resulted in a strong decrease in the viability of the detached cells. The remaining cells, however, were not affected. Usually, Accutase™ is believed to be a mild-acting enzyme that is gentle on cells and does not affect the majority of cell surface proteins, whereas trypsin is harsher and degrades most cell surface markers [25,26,27]. Accordantly, we observed a slower detachment of cells with Accutase™; however, the decreased viability was unexpected. Furthermore, it was conspicuous that the number of NGFRp75^+^ cells was always lower with trypsin-EDTA than with Accutase™. Many surface proteins and extracellular matrix components are degraded during proteolytic enzyme digestion. This can affect the detectability of surface markers and reduce cell viability, particularly that of stem cells [25]. So, while Accutase™ seemed to impair cell viability, trypsin appeared to slightly degrade NGFRp75 on the cell surface, thereby decreasing the amount of detected stem cells. Recent studies have shown that Accutase™ may also affect the expression of certain cell surface proteins and that cells need around one day of recovery time after detachment before experiments [25].

In addition to the purity of the detached DSCs, the recovery rate (or yield) is also important for evaluating the success of this method. The recovery was calculated as the ratio of the absolute number of DSCs in the detached sample to the absolute number of DSCs before detachment. Naturally, the recovery rates increased, with longer incubation times for both detachment solutions (Figure 3C and Figure S3E). However, the highest purity was associated with the second-lowest recovery, leaving almost no cells for further cultivation (Figure 3C and Figure S3E, A2: Accutase™ for 2 min). Higher recovery rates were always correlated with decreasing purity. Long cultivation periods would be necessary subsequent to the selective detachment to propagate the DSCs or to perform multiple cycles for better purity, leading to undesired high passages and making this method very time-consuming. Similarly, in a study by Kisselbach et al., multiple treatments of human primary CEC (corneal epithelial cells) cultures with trypsin-EDTA were needed due to the regrowth of fibroblasts after a couple of days, eventually leading to senescence of the desired cells. Therefore, they described this approach as simple and initially successful, yet inefficient [13]. On the contrary, for HDMEC (human dermal microvascular endothelial cells) cultures, this is an established method that is efficient in the early days of primary cell culture (first week) before fibroblasts grow too much and HDMECs adhere too strongly [14]. Considering the conditions of the DSC-fibroblast co-cultures in this study, the amount of fibroblasts might have already been too high and the adhesion of the DSCs too strong to efficiently separate the two cell types through selective detachment.

### 3.4. Separation of the Two Cell Types through Immunomagnetic Cell Sorting

DSC-fibroblast co-cultures with an average stem cell frequency below 15%, representing most primary donor cell strains, were subjected to both negative or positive selection, while also comparing EasySep™ (column-free) and MACS^®^ (automatic column-based) separation methods.

#### 3.4.1. Negative Selection

Negative selection is often the method of choice, as the cells of interest are unbound by antibodies and beads (“untouched”) [28] and thus should remain functionally unaltered [29]. Moreover, dead cells and cell debris often get labeled non-specifically and would then also be removed [30]. In contrast, negative selection is mostly less pure than positive selection methods since it is more difficult to target all unwanted cells. For this, it is necessary to know exactly what kind of cells are in the starting cell suspension, and there must be a unique surface marker for every cell type [31].

**Figure 4:**
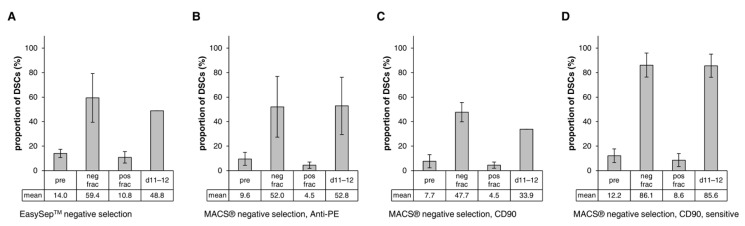
Negative selection (labeling of fibroblasts). (**A**) EasySep™ column-free negative selection with the EasySep™ Human PE Positive Selection Kit II in combination with a PE-conjugated anti-CD90 antibody (StemCell Technologies). Sample size: n = 5. (**B**) MACS^®^ automatic column-based negative selection with Anti-PE MicroBeads in combination with a PE-conjugated anti-CD90 antibody and the autoMACS^®^ Pro Separator (Miltenyi Biotec). Program: *Deplete*. Sample size: n = 3. (**C**) MACS^®^ automatic column-based negative selection with CD90 MicroBeads and the autoMACS^®^ Pro Separator (Miltenyi Biotec). Program: *Deplete*. Sample size: n = 2. (**D**) MACS^®^ automatic column-based negative selection with CD90 MicroBeads and the autoMACS^®^ Pro Separator (Miltenyi Biotec). Sensitive Program: *DepleteS*. Sample size: n = 3. Values are presented as mean ± SDs. Pre: initial sample, neg frac: DSC fraction, pos frac: fibroblast fraction, d11–12: 11–12 days of cultivation. Frequency of DSCs in the separate fractions was determined via flow cytometry analysis of NGFRp75 and CD90. Following separation, the enriched DSC fraction was cultivated in stem cell medium, and after 11–12 days the proportion of DSCs in culture was measured again via flow cytometry.

First, the EasySep™ negative selection of DSC-fibroblast co-cultures, through the depletion of fibroblasts using a CD90-PE antibody and anti-PE RapidSpheres™, was conducted. The results showed an approximately four-fold enrichment of DSCs from 14.0 ± 3.3% in the pre sample to 59.4 ± 19.8% in the negative fraction (DSC fraction) (Figure 4A), but a large number of CD90^+^ fibroblasts and sometimes also a smaller population of double-negative cells (CD90^−^/NGFRp75^−^) remained (Figure S4A, neg frac). In addition, separation was associated with a great loss of (CD90^dim^) DSCs in the positive fraction (fibroblast fraction) (10.8 ± 4.7%) and a decreasing stem cell frequency in the subsequent cultivation of the purified DSC fraction (48.8% on day 11–12) (Figure 4A and Figure S4B). The depletion of fibroblasts with the EasySep™ negative selection was not reliably reproducible, as evidenced by large variations in the results of different experiments.

The MACS^®^ approach for negative selection (labeling of fibroblasts) included two different types of MACS^®^ MicroBeads, using indirect or direct labeling. MACS^®^ negative separation via indirect labeling with Anti-PE MicroBeads in combination with a PE-conjugated anti-CD90 antibody led to a five-fold enrichment of DSCs from 9.6 ± 5.3% to 52.0 ± 24.8%, with only a minor loss of stem cells in the positive fibroblast fraction (4.5 ± 2.6%) (Figure 4B). This method also showed huge standard deviations. Again, a small population of double-negative cells (CD90^−^/NGFRp75^−^) and many fibroblasts (CD90^dim^) remained in the purified DSC fraction (Figure S4C), but on average the cultures were able to maintain the enhanced DSC frequency during subsequent cultivation for 11–12 days (52.8 ± 23.4%) (Figure 4B and Figure S4D).

The MACS^®^ negative selection through the direct labeling of fibroblasts with CD90 MicroBeads showed very similar results: the DSC frequency increased from 7.7 ± 5.4% to 47.7 ± 7.8%, and only a small amount of stem cells was separated out with the fibroblast fraction (4.5 ± 2.5%) (Figure 4C). On the contrary, the DSC proportion decreased to 33.9% on day 11–12 of cultivation (Figure 4C and Figure S4E,F). Flow cytometry analysis revealed the same distribution of cell populations as in the separation with Anti-PE MicroBeads (Figure S4C). Contrary to expectations, with both MACS^®^ negative selection procedures, many of the CD90^−^/NGFRp75^−^ cells were also found in the positive fibroblast fraction.

Good purification of DSC-fibroblast co-cultures with a lower stem cell frequency of <15% was achieved via MACS^®^ negative selection with CD90 MicroBeads (direct labeling) and a more sensitive separation program (*DepleteS*). Selection resulted in a seven-fold enrichment of DSCs from 12.2 ± 5.5% to 86.1 ± 9.8% (Figure 4D) and showed a remarkably clear separation of the different cell populations in flow cytometry analysis (Figure S4G). The other cells in the negative fraction (DSC fraction) were mostly double-negative cells (CD90^−^/NGFRp75^−^), while all CD90^+^ fibroblasts were depleted. However, a considerable amount of NGFRp75^+^/CD90^dim^ DSCs (8.6 ± 5.2%) got lost in the positive fibroblast fraction. The enriched cultures were able to maintain the high proportion of DSCs when cultivated (85.6 ± 9.5% at day 11–12) (Figure 4D and Figure S4H).

A negligible number of donor cell strains (see Figure 1) initially had or spontaneously acquired a relatively high (>30%) DSC frequency during cultivation. We assumed that purification of these cultures would be more efficient because the number of fibroblasts that had to be eliminated was smaller. Therefore, negative selection (labeling of fibroblasts) was also performed on DSC-fibroblast co-cultures with a higher proportion of stem cells. These cultures were naturally enriched or manually pre-enriched via negative selection, as described above, with intermediate cultivation.

The EasySep™ negative separation of such co-cultures (38.0 ± 2.6%) resulted in a highly enriched DSC fraction with 88.4 ± 12.0% DSCs, which may maintain the high stem cell content during further cultivation (Figure S5A,B). However, the follow-up (89.4%) included only one experiment after 28 days, meaning that the cells required considerably longer to proliferate (Figure S5A,C). Additionally, a large number of DSCs got lost in the positive fibroblast fraction (34.9 ± 10.1%).

MACS^®^ negative separation with CD90 MicroBeads of DSC-fibroblast co-cultures with a higher initial proportion of stem cells improved the DSC frequency from 54.3 ± 5.0% to 77.0 ± 6.5%, including a great loss of DSCs in the fibroblast fraction as well (49.4 ± 4.2%) (Figure S5D,E). In subsequent cultivation, the proportion of stem cells decreased to 66.7 ± 10.8% on day 11–12 (Figure S5D,F).

We showed that enrichment of DSCs with both EasySep™ negative selection and MACS^®^ negative selection was not sufficient, as it was not possible to entirely eliminate all CD90^+/dim^ fibroblasts. The negative selection approach might require multiple purification steps to completely eliminate any remaining fibroblasts [10]. Moreover, during selection, a considerable amount of DSCs got lost in the (positive) fibroblast fraction. The precise reasons for this are unknown, but could include non-specific binding, endocytosis of beads [32], or that the DSCs might not be completely CD90^−^ but rather contain a small CD90^dim^ subpopulation. Non-specific binding can occur between cells and antibodies or magnetic beads [29,33], but also dead cells and cell debris are known to be sticky and to bind non-specifically to antibodies, beads, and living cells, preventing the binding of antibodies and causing cell aggregation, thereby impairing selection efficiency [23,29]. For the MACS^®^ method, pre-incubating the samples with Basic MicroBeads to remove sticky or dead cells was ineffective (Figure S1); however, a preceding DNase digestion step and prior filtering of the cell suspension were included in the protocol. This could partially explain the lower loss of DSCs in the positive fraction as compared to the EasySep™ method. Therefore, at least a DNase digestion step and filtering (and perhaps also a dead cell removal kit) should be included in the EasySep™ protocol. Additionally, performing the incubation of cells with EasySep™ RapidSpheres™ at room temperature might favor endocytosis/internalization of the beads [34], as well as non-specific binding, and thus working at a temperature of 4 °C (equal to the MACS^®^ approach) should be considered. Further investigation is required to determine the source of the loss of (CD90^dim^) DSCs in the positive fraction and to further improve the separation efficiency.

Our results from the negative selection experiments contrast with other studies that have been able to successfully eliminate fibroblasts from endothelial cell cultures [5] or from human umbilical vein endothelial cells (HUVEC) [13] using CD90-magnetic cell sorting. However, there were some differences between the studies that might have affected the outcomes. First, we used naturally occurring co-cultures of DSCs and fibroblasts. It is unclear if stem cell medium changes the CD90 expression levels of fibroblasts and makes them less accessible for selection through CD90 antibodies. Indeed, after selection, a small population of double-negative (CD90^−^/NGFRp75^−^) cells and many CD90^+/dim^ cells remained in the purified (negative) DSC fraction, implying that these cells could not be separated based on the CD90 marker [35]. We do not know if these cells were initially present in the dermis of the skin and have been co-cultured over time, or whether these are fibroblasts that have changed their CD90 expression levels under the culture conditions in stem cell medium. This medium containing BSA might not be appropriate for CD90 expression and the proliferation of fibroblasts, which require FBS [13,15]. Identification and characterization of the CD90^−^/NGFRp75^−^ cell population would be necessary in order to eliminate it. The other groups, in contrast, experimentally mixed fibroblasts—which were cultured in their appropriate medium and thus may express CD90 differently—to the desired cell cultures right before selection to establish the separation protocol. Another important aspect to consider is the percentage of “contaminating fibroblasts” in the co-culture. While we were dealing with cultures containing on average 90% fibroblasts, the highest fibroblast contamination in both other groups reached about 50%. Therefore, an adequate comparison between these studies and our results is difficult.

#### 3.4.2. Positive Selection

Since the results from the negative selection were not satisfactory, positive selection based on NGFRp75 was performed on DSC-fibroblast co-cultures with a stem cell frequency <15%—similar to the majority of primary donor cell strains—while comparing the EasySep™ (column-free) and MACS^®^ (automatic column-based) separation methods.

The positive selection technology usually offers highly purified cells by specifically targeting the cells of interest. Compared to negative selection, it has a high yield of cells and is faster when there are multiple unwanted cell populations as it requires only one antibody [31]. However, cell debris and dead cells may also be selected due to non-specific binding [30]. The greatest disadvantage of this method is that the target cells are bound to the antibodies or other labeling agents (e.g., beads), which may affect some downstream assays and may interfere with cell viability and proliferation. Labeling of the desired cells is associated with the risk of possible artificial cell activation [22,31,32] and functional alteration [29,34].

**Figure 5:**
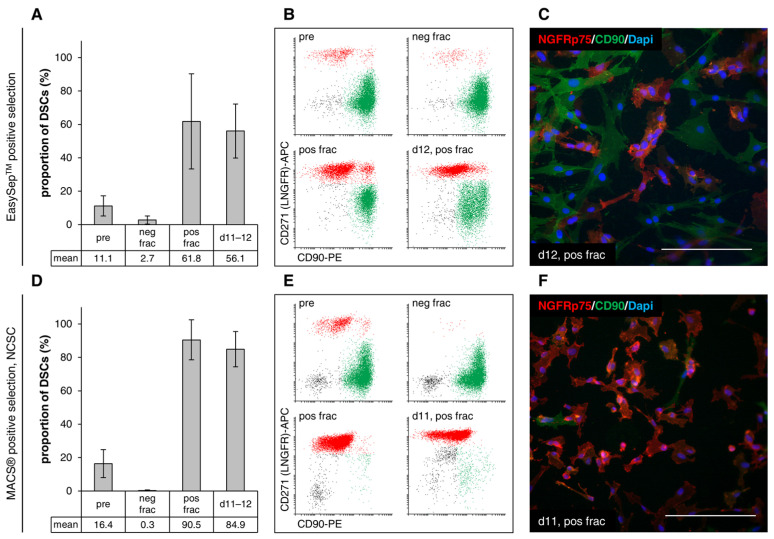
Positive selection (labeling of DSCs). (**A**–**C**) EasySep™ column-free positive selection with the EasySep™ Human CD271 Positive Selection Kit II (StemCell Technologies). Sample size: n = 6. (**D**–**F**) MACS^®^ automatic column-based positive selection with Neural Crest Stem Cell (NCSC) MicroBeads and the autoMACS^®^ Pro Separator (Miltenyi Biotec). Program: *Posseld2*. Sample size: n = 13. Values are presented as mean ± SDs. Pre: initial sample, neg frac: fibroblast fraction, pos frac: DSC fraction, d11–12: 11–12 days of cultivation. Frequency of DSCs in the separate fractions was determined via flow cytometry analysis of NGFRp75 (*y*-axis of dot plots) and CD90 (*x*-axis of dot plots). Following separation, the enriched DSC fraction was cultivated in stem cell medium. After 11–12 days, the proportion of DSCs in culture was measured again via flow cytometry, and cells were additionally stained immunohistochemically for NGFRp75 (red) and CD90 (green). Nuclei were counterstained with DAPI (blue). Scale bars: 200 µm.

EasySep™ positive selection through the labeling of DSCs via CD271 (NGFRp75) led to enriched cultures, with an average of 61.8 ± 28.5% stem cells in the positive fraction (DSC fraction) starting from 11.1 ± 6.0% in the pre sample (Figure 5A). The loss of DSCs in the negative fraction (fibroblast fraction) was minimal (2.7 ± 2.4%) (Figure 5B). After 11–12 days, on average, the DSC cultures remained enriched (56.1 ± 16.2%) (Figure 5A,C).

MACS^®^ positive selection with Neural Crest Stem Cell (NCSC) MicroBeads via NGFRp75 resulted in almost completely pure DSC cultures (90.5 ± 12.0% in positive fraction compared to 16.4 ± 8.4% in pre sample) without any loss of stem cells in the negative fraction (0.3 ± 0.3%) (Figure 5D,E). Numerous of the other remaining cells in the positive DSC fraction represented CD90^−^/NGFRp75^−^ cells, although most of them got separated off with the fibroblast fraction (Figure 5E). DSC cultures purified using this method maintained a very high stem cell frequency, averaging 84.9 ± 10.5% during cultivation for 11–12 days (Figure 5D,F).

Both positive selection procedures led to an enrichment of DSCs, yet the EasySep™ positive selection was not reliable through different experiments and was sometimes not well tolerated by the DSCs, resulting in morphological changes and poor attachment when cultivated after separation. Impaired proliferation of the stem cells (observation) led to a rapid decrease in DSC frequency and takeover of the remaining fibroblasts. It is known that magnetic particles can lead to adverse effects such as influencing cell phenotype, function [36,37], and viability [38,39,40]. This indicates that the DSCs might have been affected by the antibodies and RapidSpheres™, which they were still attached to. Reducing the amount of labeling reagents should be considered, as this is a crucial factor in positive selection [30]. With a low bead number, some target cells will be lost, but an excessive number of beads might impede proper cell growth afterwards [16]. The incorporation of a bead removal step after separation could also improve cell viability.

On the contrary, only with MACS^®^ positive selection did we obtain highly enriched DSC cultures in a reproducible manner, while this method was better tolerated by the DSCs, even though the beads remained bound to the cells, too. This could be explained by the fact that MACS^®^ MicroBeads are biodegradable. They are described to be internalized through endocytosis, followed by degradation in lysosomes within a few hours to days [41,42]. Furthermore, magnetic beads are diluted over the daughter cells through cell division in proliferating cells, whereas they can persist on the cell surface and intracellularly for up to two weeks after separation in non-dividing cells [43]. In fact, MACS^®^-purified DSCs showed better proliferation after separation than EasySep™-purified DSCs (observed as faster reached confluence and higher cell numbers after the same cultivation period), indicating that the MACS^®^ MicroBeads were diluted faster than the EasySep™ RapidSpheres™. Similarly, in previous studies where the MACS^®^ and EasySep™ technologies were compared for the isolation of monocytes and osteoprogenitor cells via positive selection, the MACS^®^ technology led to a higher isolation yield as well as a higher purity compared to EasySep™ [34,44].

Further studies would be advised to better define the influence that both technologies (EasySep™ and MACS^®^) have on the cell functionality of DSCs, especially with regard to the capability of differentiation into melanocytes. In particular, the persistence of beads on and inside the cells [43] must be carefully examined. In several studies, MACS^®^-purified cells remained highly viable and functional after positive selection [6,10]. Meyers et al. showed that human perivascular stem/stromal cells (PSC) derived through positive MACS^®^ maintained their capacity for multilineage differentiation and were even applicable for the repair of bone defects in in vivo applications [45]. Our first experiments with MACS^®^-purified DSCs showed that they retained their differentiation potential after positive selection.

Despite the high enrichment with positive selection, there were still CD90^+^ fibroblasts and double-negative (CD90^−^/NGFRp75^−^) cells found in the purified DSC fraction. The remaining cell contamination after separation was most probably attributed to direct non-specific binding between undesired cells and magnetic beads [29,33] or the stickiness of dead cells and cell debris, causing the non-specific aggregation of antibodies, beads, and living cells [23,29]. The DNase digestion step, filtering of the cell suspension prior to selection, and handling at 4 °C included in the MACS^®^ approach could be responsible for the better purification compared to the EasySep™ method and should also be included in the latter one. The use of Basic MicroBeads in the MACS^®^ separation for the removal of material binding non-specifically to magnetic beads had no effect on the separation efficiency (Figure S1).

**Figure 6:**
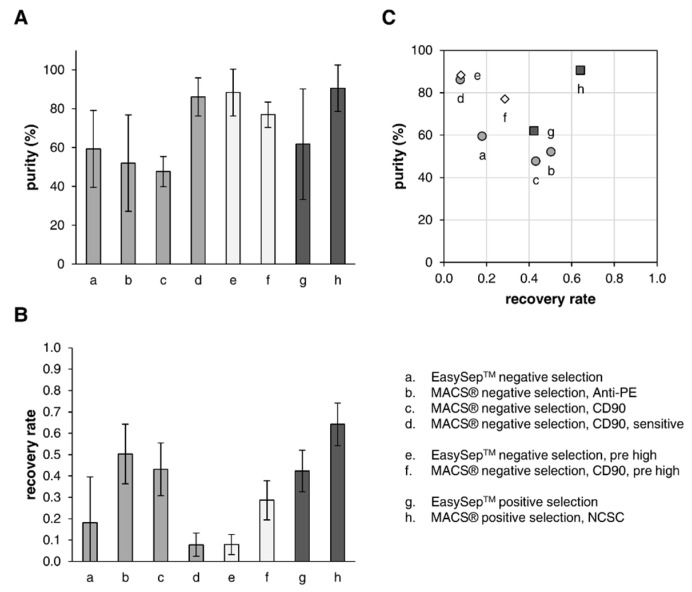
Overview of purity and recovery of DSCs after the individual selection methods. (**A**) Purity. Frequency of DSCs in the purified fraction after separation determined via flow cytometry analysis of NGFRp75. Values are presented as mean ± SDs. (**B**) Recovery rate. Ratio of the absolute number of DSCs in the purified fraction after separation to the absolute number of DSCs in the pre sample. Values are presented as mean ± SDs. (**C**) Plot of recovery rate (*x*-axis) versus purity (*y*-axis) of the individual selection methods. Sample size: a. n = 5; b. n = 3; c. n = 2; d. n = 3; e. n = 3; f. n = 2; g. n = 6; h. n = 13.

For an adequate assessment of the quality of the individual separation methods for low initial DSC frequencies, not only the purity of the DSC cultures directly after selection (Figure 6A) is important, but also the recovery rate (or yield) of the method (Figure 6B) is highly relevant. The recovery was calculated as the ratio of the absolute number of DSCs in the purified fraction to the absolute number of DSCs in the pre sample. In terms of purity, the MACS^®^ positive selection (Figure 6: h, purity 90.5 ± 12.0%, recovery 0.64 ± 0.10) through the binding of DSCs and the MACS^®^ negative selection (CD90) with a more sensitive program (Figure 6: d, 86.1 ± 9.8%, 0.08 ± 0.05) provided the best results, with the latter one not being practically applicable because of the extremely low recovery rate, leaving almost no cells for further cultivation or experiments. To obtain enough cells for downstream experiments, these purified DSCs would have to be cultured over a long period of time, causing additional costs for the expensive stem cell medium, but also bearing the risk of a decreasing DSC frequency due to some remaining fibroblasts gaining a survival advantage.

The other three methods for negative selection via labeling of fibroblasts (Figure 6: a, b, c) as well as the EasySep™ positive selection (Figure 6: g) showed comparable results for purity, with values ranging between 50–60%. However, evaluation of the recovery rate revealed clear advantages of the MACS^®^ negative selection Anti-PE (Figure 6: b, 52.0 ± 24.8%, 0.50 ± 0.14), the MACS^®^ negative selection CD90 (Figure 6: c, 47.7 ± 7.8%, 0.43 ± 0.12), and the EasySep™ positive selection (Figure 6: g, 61.8 ± 28.5%, 0.42 ± 0.10) over the EasySep™ negative selection (Figure 6: a, 59.4 ± 19.8%, 0.18 ± 0.22).

When working with higher initial DSC frequencies (≥30%), both the EasySep™ negative selection (Figure 6: e, 88.4 ± 12.0%, 0.08 ± 0.05) and the MACS^®^ negative selection (Figure 6: f, 77.0 ± 6.5%, 0.29 ± 0.09) provided a high purity but at the same time a (very) low recovery rate. The occurrence of such NGFRp75-high expressing DSC-fibroblast co-cultures is negligible. In this study, only four out of sixty-six, corresponding to six percent of the donor cell strains, were naturally enriched, indicating that this is an exception rather than the rule. Nevertheless, these results indicate that a sequential separation with two consecutive negative selections might be a good approach for achieving high purity. However, owing to the low output of negative selection, an intermediate cultivation of multiple weeks for the propagation of cells between the two separations would be necessary.

By plotting the recovery rate versus purity (Figure 6C), the MACS^®^ positive selection (Figure 6: h) stood out as the best option to enrich DSCs in cell culture due to both the highest purity and also the best recovery rate.

It is necessary to find the appropriate balance between purity and recovery. Typically, the optimization of one parameter results in the impairment of the other [30]. Regarding negative selection, increasing the amount of antibody and beads as well as the incubation period might improve the targeting of fibroblasts, increasing the purity, but is also likely to enhance non-specific binding of DSCs, thereby decreasing recovery. Our results showed that already a considerable number of DSCs got separated out with the fibroblast fraction, and to a greater extent in the EasySep™ approach, leading to the poor recovery rate of this method. Thus, increasing the labeling reagents would only be an option for MACS^®^ negative selection. By contrast, for the negative selection approaches showing high purity (Figure 6: d, e, f), the associated low recovery rates may be improved through a reduction in the amount of labeling reagents or the incubation times, but would probably be accompanied by a decrease in purity. Regarding positive selection, reducing the incubation times may improve purity but might decrease recovery [30].

The separation efficiency of immunomagnetic cell sorting is affected by multiple factors, including the binding of antibodies and beads to the cells, the magnetic susceptibility of the beads, and the strength of the magnetic field [30,31]. One explanation for the divergences regarding viability and proliferation of enriched DSCs following positive separation could be differences in the beads used in both technologies. Column-free technology often uses micro-sized paramagnetic beads ranging typically from 0.5 to 5 µm in diameter [32]. The EasySep™ selection system uses bispecific tetrameric antibody complexes (TACs, included in the selection cocktail) that connect the (labeled) target cells to the dextran-coated EasySep™ RapidSpheres™. The manufacturer StemCell Technology does not provide any information about the exact size of their paramagnetic particles. In the literature, however, values ranging from 150 nm [32,43] to 200 nm [22] have been reported. The MACS^®^ approach from Miltenyi Biotec, by contrast, relies on nano-sized superparamagnetic MACS^®^ MicroBeads with a size of approximately 50 nm that are smaller than any other commercially available immunomagnetic beads for cell isolation [41]. These MACS^®^ MicroBeads are biodegradable, non-toxic, and they are said to be compatible with any downstream application and to not affect cell viability and proliferation [28].

Since the column-free technology (EasySep™) uses weak magnetic fields, extensive labeling and/or large beads are required. This not only promotes non-specific labeling but also leads to epitope saturation, which could interfere with downstream applications such as flow cytometry. More importantly, this affects cell viability and cell characteristics, resulting in the activation of cells [32,46] and biological alterations [34]. Because of the stronger magnetic field in the column-based approach (MACS^®^), minimal labeling and smaller beads (nano-sized) are sufficient, thereby resulting in less non-specific labeling and no epitope blocking. Due to minimal cell stress and preservation of cell functionality, column-based methods are usually well tolerated by the target cells [45].

Taken together, each technology has several advantages and disadvantages, which can be confirmed after this study and are listed in Table 1 [47,48]. For time-sensitive molecular analyses, such as gene expression profiling or signal transduction assays, the MACS^®^ purification method is more suitable.

## 4. Conclusions

There is increasing evidence that in human skin, neural crest-derived DSCs might be involved in (UV-induced) melanomagenesis. DSCs isolated from human foreskin are suitable for studying the underlying processes. Because primary DSC cultures contain a considerable amount of contaminating fibroblasts, purification of these DSC-fibroblast co-cultures is an urgent prerequisite for certain investigations. Using different approaches, we demonstrated that Geneticin and selective detachment to eliminate fibroblasts are not suitable methods for the enrichment of DSCs. Negative selection approaches were suboptimal and require further optimization. Altogether, we propose positive selection via MACS^®^ technology as the method of choice for the purification of DSCs from primary cell cultures. When assessing multiple parameters, such as purity, recovery rate, and proliferation ability after separation, this approach was perceived to be superior to all other methods examined in this study. The purification and expansion of enriched DSCs in vitro is a crucial step forward in order to examine the effects of UV radiation on their capability to differentiate into melanocytes, as well as to examine genetic and epigenetic alterations. Thus, enriched DSCs represent a novel and unique model to study their potential relevance in the genesis of malignant melanoma.

## Figures and Tables

**Table 1:** Comparison of EasySep™ column-free and MACS^®^ automatic column-based selection technology.

EasySep™ Column-Free	MACS^®^ Automatic Column-Based
- considerably cheaper - minimum space requirements - lower risk of sample loss - time-consuming - labor-intensive - lower recovery, more loss of cells - success is strongly dependent on the handling of the executing person - weak magnetic field - thereby extensive labeling and/or larger beads required [31] ○ leads to epitope saturation ○ promotes non-specific labeling ○ impacts the viability and cell characteristics ○ can lead to activation of cells [32,46] and biological alteration [34]	- more expensive - device takes up a lot of space in the laminar flow hood - samples can get lost through clogging of columns or intake of air bubbles - new columns are needed periodically - excessive washing necessary to avoid contaminations - fast protocol - easy handling [45] - better separation - standardized separation, well reproducible - strong magnetic field [28,32] - thereby minimal labeling and smaller beads (nano-sized) are sufficient [31] ○ no epitope blocking [45] ○ less non-specific labeling ○ little cell stress ○ preservation of cell functionality ○ well tolerated by target cells

## Data Availability

The datasets generated and/or analyzed during this study are available from the corresponding author upon reasonable request.

## Supplementary Materials

The following supporting information can be downloaded at: https://www.mdpi.com/article/10.3390/cells12060949/s1, Figure S1: Effect of prior usage of Basic MicroBeads on immunomagnetic cell sorting; Figure S2: Geneticin treatment of DSC-fibroblast co-cultures; Figure S3: Selective detachment of DSC-fibroblast co-cultures with <5% or >30% stem cells; Figure S4: Negative selection (labeling of fibroblasts); Figure S5: Negative selection (labeling of fibroblasts) of cell cultures with higher initial DSC frequency.

## References

[1] Hoerter J.D., Bradley P., Casillas A., Chambers D., Denholm C., Johnson K., Weiswasser B. (2012). Extrafollicular dermal melanocyte stem cells and melanoma. Stem. Cells Int..

[2] Zabierowski S.E., Fukunaga-Kalabis M., Li L., Herlyn M. (2011). Dermis-derived stem cells: A source of epidermal melanocytes and melanoma?. Pigment. Cell Melanoma Res..

[3] Li L., Fukunaga-Kalabis M., Yu H., Xu X., Kong J., Lee J.T., Herlyn M. (2010). Human dermal stem cells differentiate into functional epidermal melanocytes. J. Cell Sci..

[4] Mhamdi-Ghodbani M., Starzonek C., Degenhardt S., Bender M., Said M., Greinert R., Volkmer B. (2021). UVB damage response of dermal stem cells as melanocyte precursors compared to keratinocytes, melanocytes, and fibroblasts from human foreskin. J. Photochem. Photobiol. B.

[5] Saalbach A., Aust G., Haustein U.F., Herrmann K., Anderegg U. (1997). The fibroblast-specific MAb AS02: A novel tool for detection and elimination of human fibroblasts. Cell Tissue Res..

[6] Peng K., Sant D., Andersen N., Silvera R., Camarena V., Pinero G., Graham R., Khan A., Xu X.M., Wang G. (2020). Magnetic separation of peripheral nerve-resident cells underscores key molecular features of human Schwann cells and fibroblasts: An immunochemical and transcriptomics approach. Sci. Rep..

[7] Calderón-Martínez D., Garavito Z., Spinel C., Hurtado H. (2002). Schwann cell-enriched cultures from adult human peripheral nerve: A technique combining short enzymatic dissociation and treatment with cytosine arabinoside (Ara-C). J. Neurosci. Methods.

[8] Lehmann H.C., Chen W., Mi R., Wang S., Liu Y., Rao M., Höke A. (2012). Human Schwann cells retain essential phenotype characteristics after immortalization. Stem. Cells Dev..

[9] Gledhill K., Guo Z., Umegaki-Arao N., Higgins C.A., Itoh M., Christiano A.M. (2015). Melanin Transfer in Human 3D Skin Equivalents Generated Exclusively from Induced Pluripotent Stem Cells. PLoS ONE.

[10] Li S., Zenkel M., Kruse F.E., Giessl A., Schlotzer-Schrehardt U. (2022). Identification, Isolation, and Characterization of Melanocyte Precursor Cells in the Human Limbal Stroma. Int. J. Mol. Sci..

[11] Hsiao F.S., Cheng C.C., Peng S.Y., Huang H.Y., Lian W.S., Jan M.L., Fang Y.T., Cheng E.C., Lee K.H., Cheng W.T. (2010). Isolation of therapeutically functional mouse bone marrow mesenchymal stem cells within 3 h by an effective single-step plastic-adherent method. Cell Prolif..

[12] Tomlinson M.J., Tomlinson S., Yang X.B., Kirkham J. (2013). Cell separation: Terminology and practical considerations. J. Tissue Eng..

[13] Kisselbach L., Merges M., Bossie A., Boyd A. (2009). CD90 Expression on human primary cells and elimination of contaminating fibroblasts from cell cultures. Cytotechnology.

[14] Henrot P., Laurent P., Levionnois E., Leleu D., Pain C., Truchetet M.E., Cario M. (2020). A Method for Isolating and Culturing Skin Cells: Application to Endothelial Cells, Fibroblasts, Keratinocytes, and Melanocytes From Punch Biopsies in Systemic Sclerosis Skin. Front. Immunol..

[15] Komiyama T., Nakao Y., Toyama Y., Asou H., Vacanti C.A., Vacanti M.P. (2003). A novel technique to isolate adult Schwann cells for an artificial nerve conduit. J. Neurosci. Methods.

[16] Bourland J., Mayrand D., Tremblay N., Moulin V.J., Fradette J., Auger F.A. (2019). Isolation and Culture of Human Dermal Microvascular Endothelial Cells. Methods Mol. Biol..

[17] Liao X., Makris M., Luo X.M. (2016). Fluorescence-activated Cell Sorting for Purification of Plasmacytoid Dendritic Cells from the Mouse Bone Marrow. J. Vis. Exp..

[18] Manohar S.M., Shah P., Nair A. (2021). Flow cytometry: Principles, applications and recent advances. Bioanalysis.

[19] Polisetti N., Schlotzer-Schrehardt U., Reinhard T., Schlunck G. (2020). Isolation and enrichment of melanocytes from human corneal limbus using CD117 (c-Kit) as selection marker. Sci. Rep..

[20] Willemsen M., Luiten R.M., Teunissen M.B.M. (2020). Instant isolation of highly purified human melanocytes from freshly prepared epidermal cell suspensions. Pigment. Cell Melanoma Res..

[21] Sobiepanek A., Kowalska P.D., Soszyńska M., Ścieżyńska A. (2020). Implementation of Geneticin in the in vitro cell culture and in vivo studies. Rev. Res. Cancer Treat..

[22] Zborowski M., Chalmers J.J. (2005). Magnetic cell sorting. Methods Mol. Biol..

[23] Ravelo K.M., Andersen N.D., Monje P.V. (2018). Magnetic-Activated Cell Sorting for the Fast and Efficient Separation of Human and Rodent Schwann Cells from Mixed Cell Populations. Methods Mol. Biol..

[24] Moulin V.J., Mayrand D., Laforce-Lavoie A., Larochelle S., Genest H. (2011). In Vitro Culture Methods of Skin Cells for Optimal Skin Reconstruction by Tissue Engineering. Regen. Med. Tissue Eng.-Cells Biomater..

[25] Lai T.Y., Cao J., Ou-Yang P., Tsai C.Y., Lin C.W., Chen C.C., Tsai M.K., Lee C.Y. (2022). Different methods of detaching adherent cells and their effects on the cell surface expression of Fas receptor and Fas ligand. Sci. Rep..

[26] Skog M., Sivler P., Steinvall I., Aili D., Sjoberg F., Elmasry M. (2019). The Effect of Enzymatic Digestion on Cultured Epithelial Autografts. Cell Transpl..

[27] Quan Y., Yan Y., Wang X., Fu Q., Wang W., Wu J., Yang G., Ren J., Wang Y. (2012). Impact of cell dissociation on identification of breast cancer stem cells. Cancer Biomark..

[28] Miltenyi S., Müller W., Weichel W., Radbruch A. (1990). High gradient magnetic cell separation with MACS. Cytometry.

[29] Moore D.K., Motaung B., du Plessis N., Shabangu A.N., Loxton A.G. (2019). Isolation of B-cells using Miltenyi MACS bead isolation kits. PLoS ONE.

[30] Peters C.E., Woodside S.M., Eaves A.C. (2005). Isolation of subsets of immune cells. Methods Mol. Biol..

[31] Frenea-Robin M., Marchalot J. (2022). Basic Principles and Recent Advances in Magnetic Cell Separation. Magnetochemistry.

[32] Grützkau A., Radbruch A. (2010). Small but mighty: How the MACS-technology based on nanosized superparamagnetic particles has helped to analyze the immune system within the last 20 years. Cytometry A.

[33] Chalmers J.J., Xiong Y., Jin X., Shao M., Tong X., Farag S., Zborowski M. (2010). Quantification of non-specific binding of magnetic micro- and nanoparticles using cell tracking velocimetry: Implication for magnetic cell separation and detection. Biotechnol. Bioeng..

[34] Marques G.S., Silva Z., Videira P.A. (2018). Antitumor Efficacy of Human Monocyte-Derived Dendritic Cells: Comparing Effects of two Monocyte Isolation Methods. Biol. Proced. Online.

[35] Jiang D., Rinkevich Y. (2018). Defining Skin Fibroblastic Cell Types Beyond CD90. Front. Cell Dev. Biol..

[36] Farrell E., Wielopolski P., Pavljasevic P., van Tiel S., Jahr H., Verhaar J., Weinans H., Krestin G., O’Brien F.J., van Osch G. (2008). Effects of iron oxide incorporation for long term cell tracking on MSC differentiation in vitro and in vivo. Biochem. Biophys. Res. Commun..

[37] Meinhardt K., Kroeger I., Abendroth A., Müller S., Mackensen A., Ullrich E. (2012). Influence of NK cell magnetic bead isolation methods on phenotype and function of murine NK cells. J. Immunol. Methods.

[38] Mahmoudi M., Azadmanesh K., Shokrgozar M.A., Journeay W.S., Laurent S. (2011). Effect of nanoparticles on the cell life cycle. Chem. Rev..

[39] Shen M.J., Olsthoorn R.C.L., Zeng Y., Bakkum T., Kros A., Boyle A.L. (2021). Magnetic-Activated Cell Sorting Using Coiled-Coil Peptides: An Alternative Strategy for Isolating Cells with High Efficiency and Specificity. ACS Appl. Mater Interfaces.

[40] Van de Walle A., Perez J.E., Abou-Hassan A., Hémadi M., Luciani N., Wilhelm C. (2020). Magnetic nanoparticles in regenerative medicine: What of their fate and impact in stem cells?. Mater. Today Nano.

[41] Müller P., Gaebel R., Lemcke H., Wiekhorst F., Hausburg F., Lang C., Zarniko N., Westphal B., Steinhoff G., David R. (2017). Intramyocardial fate and effect of iron nanoparticles co-injected with MACS(^®^) purified stem cell products. Biomaterials.

[42] Müller P., Gaebel R., Lemcke H., Steinhoff G., David R. (2017). Data on the fate of MACS^®^ MicroBeads intramyocardially co-injected with stem cell products. Data Brief..

[43] Laghmouchi A., Hoogstraten C., Falkenburg J.H.F., Jedema I. (2020). Long-term in vitro persistence of magnetic properties after magnetic bead-based cell separation of T cells. Scand J. Immunol..

[44] Olbrich M., Rieger M., Reinert S., Alexander D. (2012). Isolation of osteoprogenitors from human jaw periosteal cells: A comparison of two magnetic separation methods. PLoS ONE.

[45] Meyers C.A., Xu J., Zhang L., Chang L., Wang Y., Asatrian G., Ding C., Yan N., Zou E., Broderick K. (2019). Skeletogenic Capacity of Human Perivascular Stem Cells Obtained Via Magnetic-Activated Cell Sorting. Tissue Eng. Part A.

[46] Øren A., Husebø C., Iversen A.C., Austgulen R. (2005). A comparative study of immunomagnetic methods used for separation of human natural killer cells from peripheral blood. J. Immunol. Methods.

[47] Miltenyi Biotec Magnetic Cell Separation. https://www.miltenyibiotec.com/DE-en/resources/macs-handbook/macs-technologies/cell-separation/magnetic-cell-separation.html.

[48] Miltenyi Biotec MACS® Cell Separation, Select the Best Brochure. https://www.miltenyibiotec.com/_Resources/Persistent/b5349effdd595b72195e588aff033be3e24706bd/IM0020021.pdf.

