# Supplementary material for: Enrichment of Human Dermal Stem Cells from Primary Cell Cultures through the Elimination of Fibroblasts
Source: Cells. 2023 Mar 21;12(6):949. doi: 10.3390/cells12060949 (PMC10047019; doi:10.3390/cells12060949)
Supplement: Supplementary file 1 [file cells-12-00949-s001.zip › cells-2203041-supplementary.pdf]

Supplementary Materials

Enrichment of Human Dermal Stem Cells from Primary Cell Cultures through Elimination of Fibroblasts

Christin Starzonek [1†], Mouna Mhamdi-Ghodbani [1†], Stefan Henning [1], Marc Bender [1], Sarah Degenhardt [1], I-Peng Chen [1], Mohamed Said [2], Rüdiger Greinert [1] and Beate Volkmer [1,*]

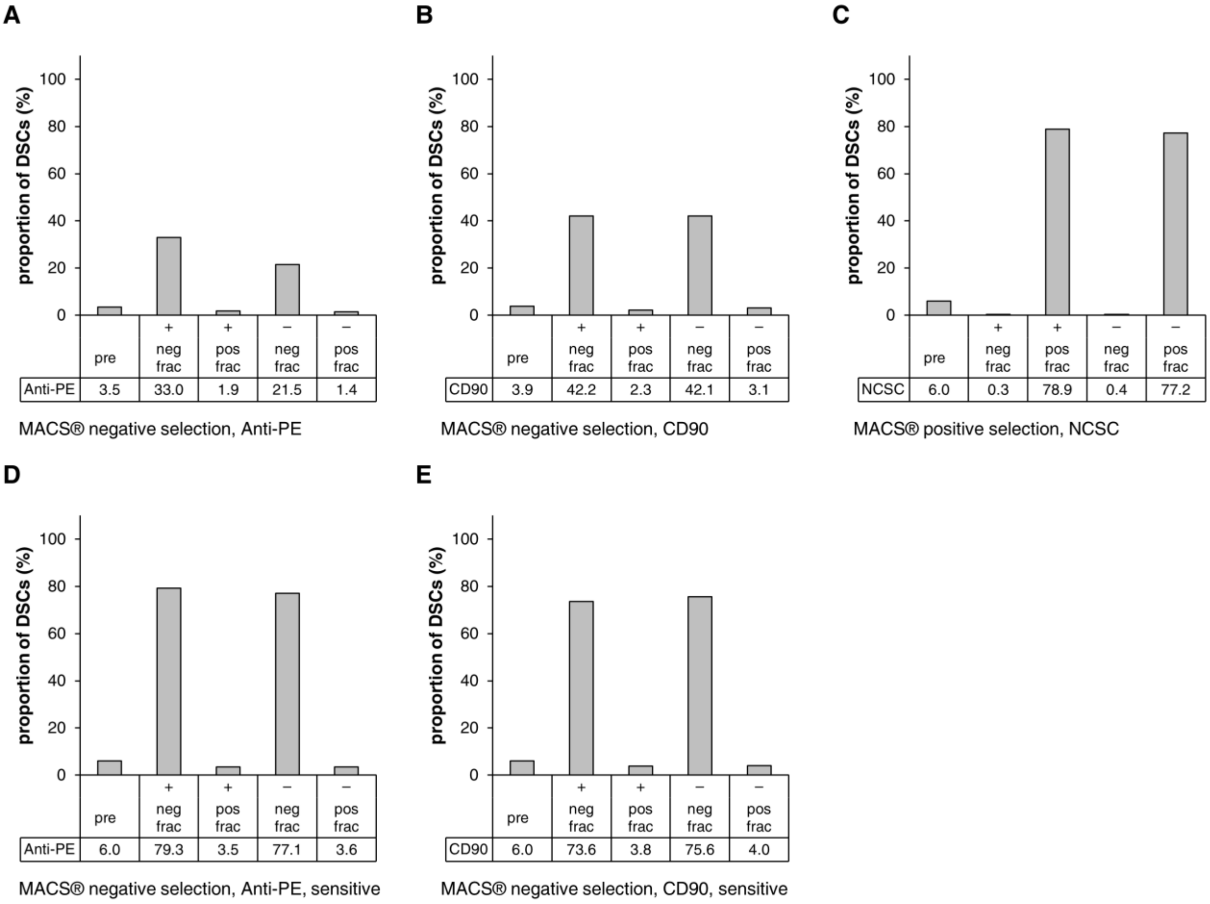

Figure S1. Effect of prior usage of Basic MicroBeads on immunomagnetic cell sorting. Comparison of separation results with prior incubation with Basic MicroBeads (+) and without usage of Basic MicroBeads (-). **(A)** MACS® automatic column-based negative selection with Anti-PE MicroBeads in combination with a PE-conjugated anti-CD90 antibody and the autoMACS® Pro Separator (Miltenyi Biotec). Program *Deplete*. Sample size: n=1. **(B)** MACS® automatic column-based negative selection with CD90 MicroBeads and the autoMACS® Pro Separator (Miltenyi Biotec). Program *Deplete*. Sample size: n=1. **(C)** MACS® automatic column-based positive selection with Neural Crest Stem Cell (NCSC) MicroBeads and the autoMACS® Pro Separator (Miltenyi Biotec). Program *Posseld2*. Sample size: n=1. **(D)** MACS® automatic column-based negative selection with Anti-PE MicroBeads in combination with a PE-conjugated anti-CD90 antibody and the autoMACS® Pro Separator (Miltenyi Biotec). Sensitive Program *DepleteS*. Sample size: n=1. **(E)** MACS® automatic column-based negative selection with CD90 MicroBeads and the autoMACS® Pro Separator (Miltenyi Biotec). Sensitive Program *DepleteS*. Sample size: n=1. Pre: initial sample, neg frac: unlabeled fraction, pos frac: labeled fraction. Frequency of DSCs in the separate fractions was determined by flow cytometry analysis of NGFRp75 and CD90.

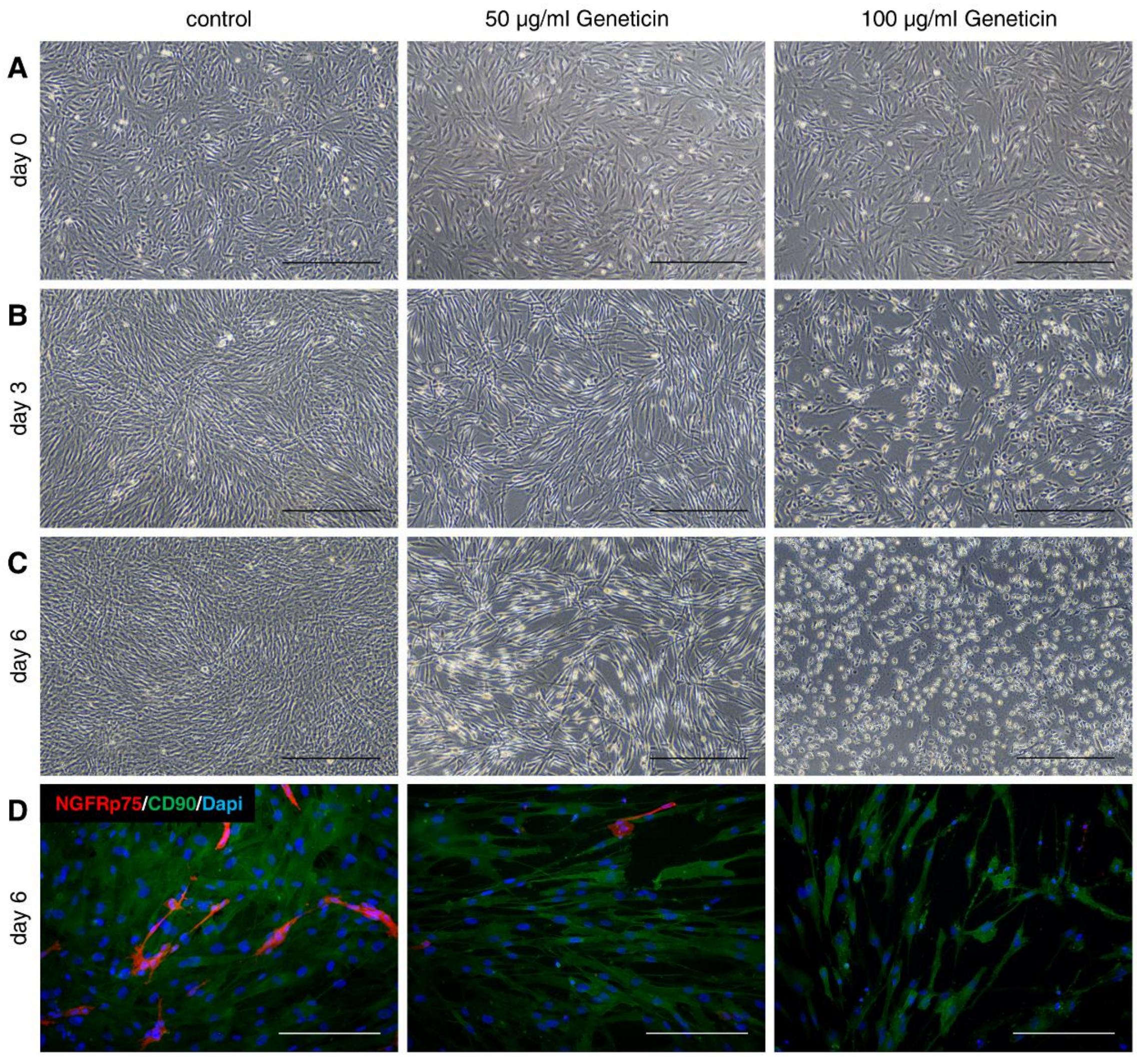

Figure S2. Geneticin treatment of DSC-fibroblast co-cultures. Dermal cells were cultured in StemPro hESC medium without Geneticin, with 50 µg/mL or 100 µg/mL Geneticin for two days. Cell growth was monitored for a total of six days and recorded on day 0 **(A)**, day 3 **(B),** and day 6 **(C)**. Cells were finally stained for the neural crest stem cell marker NGFRp75 (red) and fibroblast marker CD90 (green) on day 6 **(D)**. Nuclei were counterstained with DAPI (blue). Scale bars: (A–C) 500 µm, (D) 200 µm.

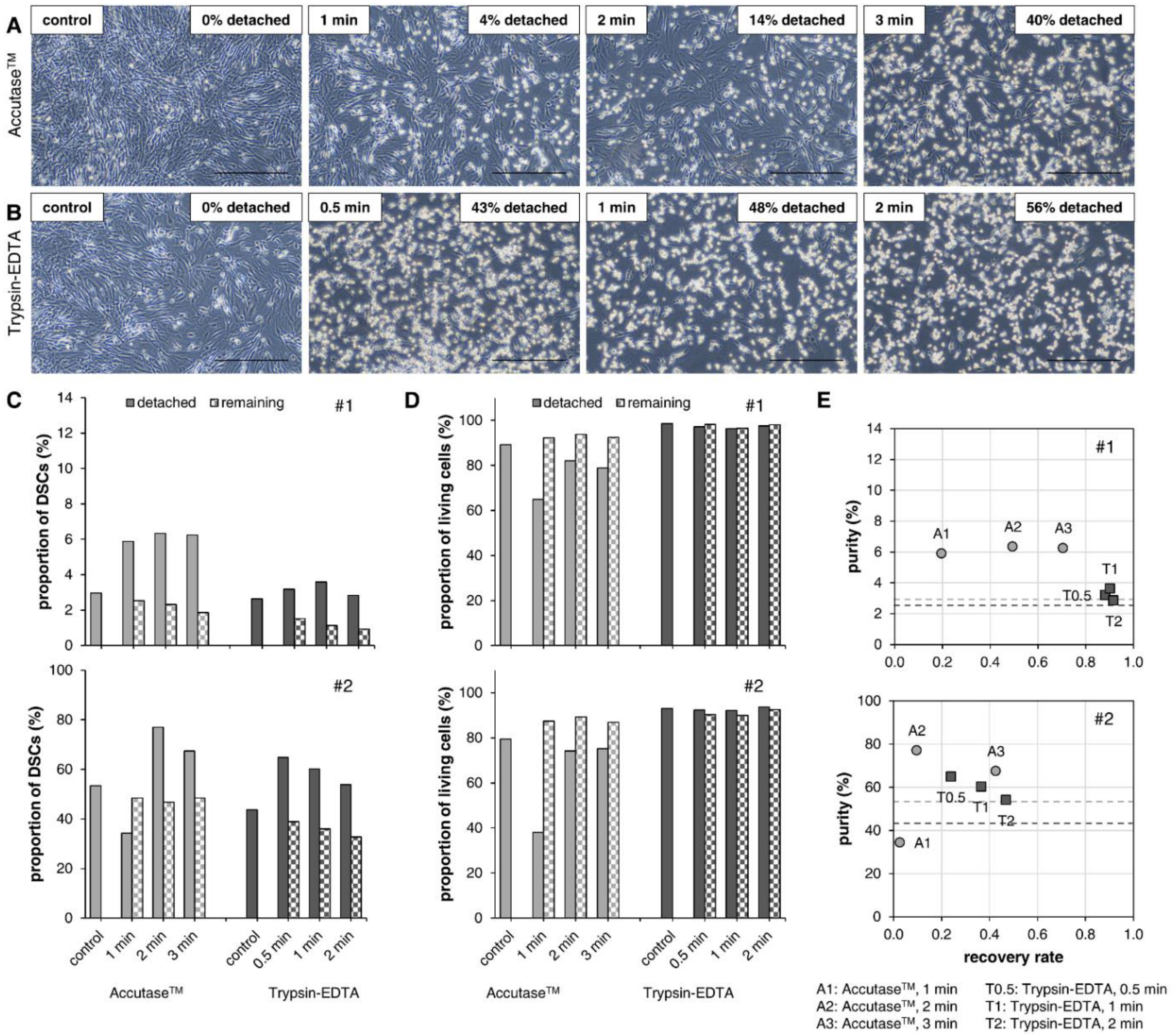

Figure S3. Selective detachment of DSC-fibroblast co-cultures with <5% or >30% stem cells. Dermal cells were incubated with Accutase™ for 1, 2, or 3 min or with trypsin-EDTA for 0.5, 1, or 2 min, and detached cells were collected. Light microscopy of the remaining cells at the different time points of detachment with Accutase™ **(A)** or trypsin-EDTA **(B)** with the percentage of detached total cells. Scale bar: 500 µm. **(C)** Frequency of DSCs in samples measured using NGFRp75 staining. **(D)** Viability of total cells examined with propidium iodide. **(E)** Plot of recovery rate (x-axis) versus purity (y-axis) of detached cells at the individual incubation times. Purity: frequency of DSCs. Recovery rate: ratio of the absolute number of DSCs in the detached sample to the absolute number of DSCs before detachment. Dotted lines indicate the DSC frequency of control cells detached with Accutase™ (light gray) or trypsin-EDTA (dark gray). Results from the other two donor cell strains.

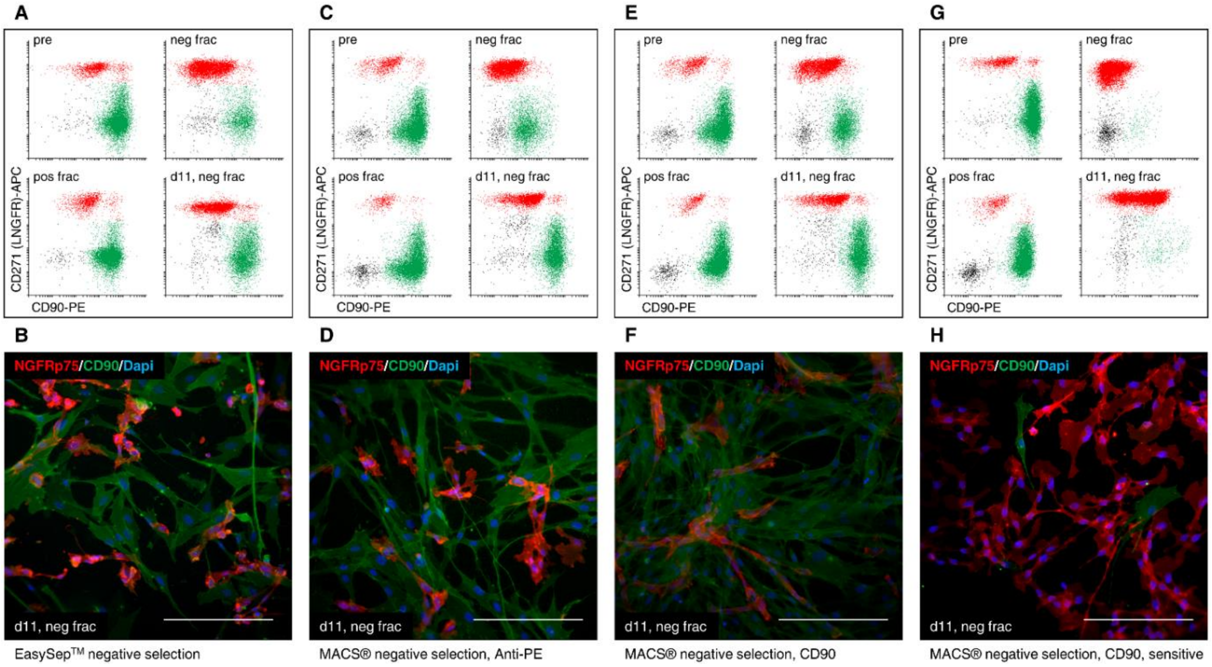

Figure S4. Negative selection (labeling of fibroblasts). **(A–B)** EasySep™ column-free negative selection with the EasySep™ Human PE Positive Selection Kit II in combination with a PE-conjugated anti-CD90 antibody (StemCell Technologies). **(C–D)** MACS® automatic column-based negative selection with Anti-PE MicroBeads in combination with a PE-conjugated anti-CD90 antibody and the autoMACS® Pro Separator (Miltenyi Biotec). Program *Deplete*. **(E–F)** MACS® automatic column-based negative selection with CD90 MicroBeads and the autoMACS® Pro Separator (Miltenyi Biotec). Program *Deplete*. **(G–H)** MACS® automatic column-based negative selection with CD90 MicroBeads and the autoMACS® Pro Separator (Miltenyi Biotec). Sensitive Program *DepleteS*. Pre: initial sample, neg frac: DSC fraction, pos frac: fibroblast fraction, d11–12: 11–12 days of cultivation. Frequency of DSCs in the separate fractions was determined by flow cytometry analysis of NGFRp75 (y-axis of dot plots) and CD90 (x-axis of dot plots) **(A+C+E+G)**. Following separation, the enriched DSC fraction was cultivated in stem cell medium. After 11–12 days, the proportion of DSCs in culture was measured again via flow cytometry, and cells were additionally stained immunohistochemically **(B+D+F+H)** for NGFRp75 (red) and CD90 (green). Nuclei were counterstained with DAPI (blue). Scale bars: 200 µm.

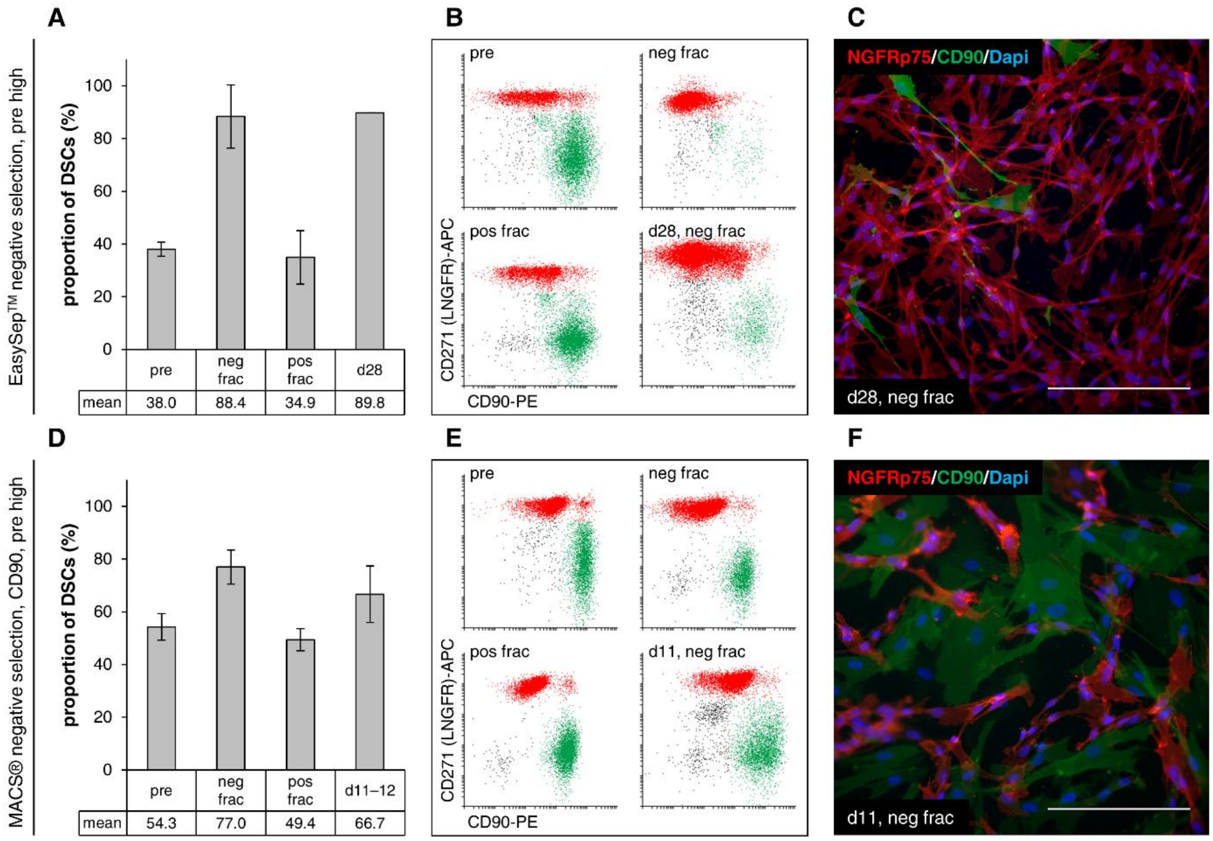

Figure S5. Negative selection (labeling of fibroblasts) of cell cultures with higher initial DSC frequency. **(A–C)** EasySep™ column-free negative selection with the EasySep™ Human PE Positive Selection Kit II in combination with a PE-conjugated anti-CD90 antibody (StemCell Technologies) in cell cultures with higher initial DSC frequencies. Sample size: n=3. **(D–F)** MACS® automatic column-based negative selection with CD90 MicroBeads and the autoMACS® Pro Separator (Miltenyi Biotec) in cell cultures with higher initial DSC frequencies. Program *Deplete*. Sample size: n=2. Values are presented as mean ± SD. Pre: initial sample, neg frac: DSC fraction, pos frac: fibroblast fraction, d28/d11–12: 28 or 11–12 days of cultivation. Frequency of DSCs in the separate fractions was determined by flow cytometry analysis of NGFRp75 (y-axis of dot plots) and CD90 (x-axis of dot plots). Following separation, the enriched DSC fraction was cultivated in stem cell medium. After 11–12 or 28 days, the proportion of DSCs in culture was measured again via flow cytometry, and cells were additionally stained immunohistochemically for NGFRp75 (red) and CD90 (green). Nuclei were counterstained with DAPI (blue). Scale bars: 200 µm.
